# Histone demethylase KDM5A promotes tumorigenesis of osteosarcoma tumor

**DOI:** 10.1038/s41420-020-00396-7

**Published:** 2021-01-12

**Authors:** Daohu Peng, Birong Lin, Mingzhong Xie, Ping Zhang, QingXi Guo, Qian Li, Qinwen Gu, Sijin Yang, Li Sen

**Affiliations:** 1 Hospital (T.C.M) Affiliated to Southwest Medical University, 182 Chunhui Road, Longmatan District, 64600 Luzhou City, Sichuan P. R. China; 2 The affiliated hospital of Southwest Medical University, 25 Taiping Street, Jiangyang District, 646015 Luzhou City, Sichuan P. R. China

**Keywords:** Sarcoma, Tumour biomarkers

## Abstract

Osteosarcoma is a primary bone malignancy with a high rate of recurrence and poorer prognosis. Therefore, it is of vital importance to explore novel prognostic molecular biomarkers and targets for more effective therapeutic approaches. Previous studies showed that histone demethylase KDM5A can increase the proliferation and metastasis of several cancers. However, the function of KDM5A in the carcinogenesis of osteosarcoma is not clear. In the current study, KDM5A was highly expressed in osteosarcoma than adjacent normal tissue. Knockdown of KDM5A suppressed osteosarcoma cell proliferation and induced apoptosis. Moreover, knockdown of KDM5A could increase the expression level of P27 (cell-cycle inhibitor) and decrease the expression of Cyclin D1. Furthermore, after knockout of KDM5A in osteosarcoma cells by CRISPR/Cas9 system, the tumor size and growth speed were inhibited in tumor-bearing nude mice. RNA-Seq of KDM5A-KO cells indicated that interferon, epithelial–mesenchymal transition (EMT), IL6/JAK/STAT3, and TNF-α/NF-κB pathway were likely involved in the regulation of osteosarcoma cell viability. Taken together, our research established a role of KDM5A in osteosarcoma tumorigenesis and progression.

## Introduction

Osteosarcoma was one of the most common primary malignancies arising from bone and affects primarily children, adolescents, and young adults^1^. Unfortunately, although modern treatment protocols combined with chemotherapy, surgery, and radiotherapy, the metastasis and recurrence of the osteosarcoma contributed mainly to the poor prognosis of patients^2^. Uncontrollable cell proliferation was one of the main factors in the carcinogenesis and development of osteosarcoma^3^. Therefore, exploring the novel molecular mechanism of tumor growth could help develop a new therapeutic intervention to improve overall outcome in patients with malignant osteosarcoma^3^.

KDM5A was originally identified as a transcriptional repressor of pRB (protein of *retinoblastoma, RB1*) and played a role in differentiation of neural progenitor cells^4–6^. Mutations in tumor suppressor genes, as *RB1*^7,8^ and p53 gene (*TP53)*, were strongly associated with human osteosarcoma^9^. Misregulation of KDM5A contributed to the pathogenesis of lung and gastric cancers^10^. KDM5A has been reported to be highly expressed in ovarian cancer tissues and especially in SKOV3/paclitaxel (PTX) cells, which were resistant to PTX^11^. KDM5A was also considered as a potential therapeutic target in small-cell lung cancer^12^, glioblastoma (GBM)^13,14^, hepatocellular carcinoma^15^, breast cancer^16^, and renal cell carcinoma^17^. However, the expression of KDM5A and its clinical significance in osteosarcoma remains unclear.

In the present work, we examined the expression of KDM5A among human osteosarcoma tissues and its relationship with the clinical characteristic patients. Moreover, the potential role of KDM5A on the proliferation of osteosarcoma was investigated. Finally, this study provided a deeper understanding of the molecular mechanism of KDM5A underlying the osteosarcoma progression.

## Results

### KDM5A was upregulated in malignant osteosarcoma tissues

**Fig. 1:**
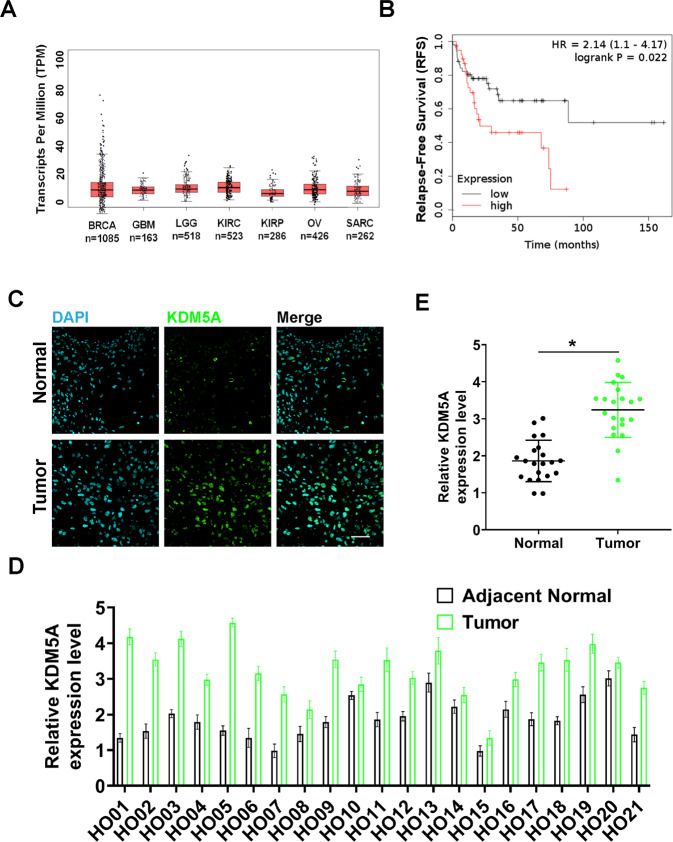
KDM5A overexpression was associated with recurrence-free survival and upregulated in human osteosarcoma carcinoma tissues. **A** KDM5A shows similar higher mRNA expression in sarcoma (SARC) tumors compared to other tumors in a publicly available website (http://gepia.cancer-pku.cn/). BRCA: breast-invasive carcinoma; GBM: glioblastoma multiforme; LGG: brain lower-grade glioma; KIRC: kidney renal clear-cell carcinoma; KIBP: kidney renal papillary cell carcinoma; OV: ovarian serous cystadenocarcinoma; SARC: sarcoma. **B** Kaplan–Meier curve showing recurrence-free survival (RFS) in SARC patients with autoselect best cutoff. The plot was generated using a publicly available website (http://kmplot.com) from the data of TCGA. **C** Immunohistochemistry (IHC) staining was performed to evaluate KDM5A expression in 21 paired osteosarcoma and adjacent normal tissues. The IHC images were captured using confocal fluorescence microscope (Olympus, FV3000, Japan). Scale bar: 50 μm. **D** Relative KDM5A expression in osteosarcoma and adjacent normal tissues was measured by calculating the integrated density (IntDen)/area (mean gray value) using ImageJ 1.52a. Expression levels were adjusted to DAPI. **E** Relative KDM5A expression was compared between osteosarcoma and adjacent normal tissues. Three independent experiments were performed, the differences between groups were assessed using unpaired *t* test for continuous variables and analysis of variance (ANOVA), and the *p*-value and the asterisk (*: *p* < 0.05) indicated a significant change compared with the adjacent normal group.

**Fig. 2:**
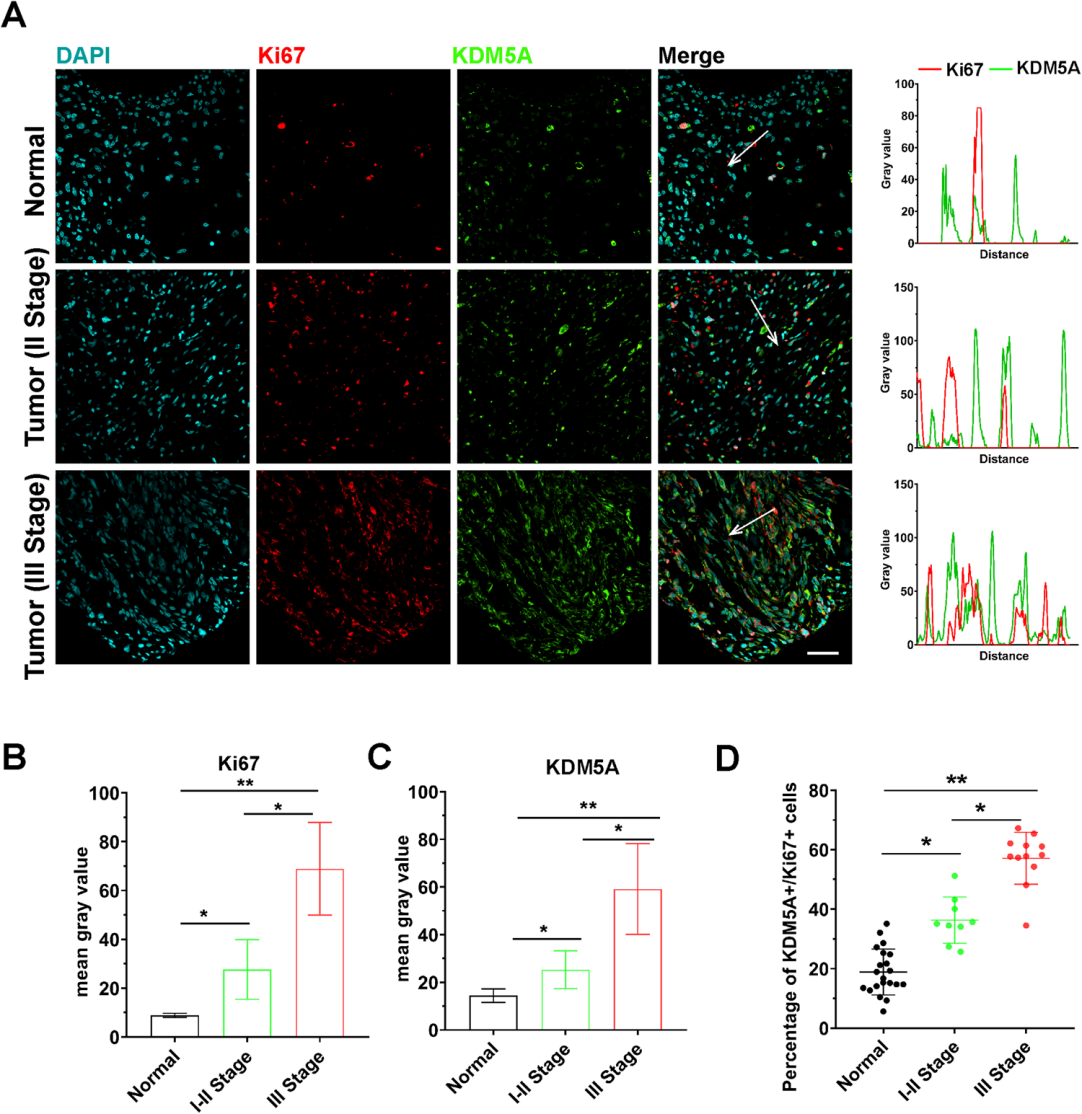
KDM5A stimulated proliferation in osteosarcoma carcinoma. **A** IHC staining of endogenous Ki67 and KDM5A in osteosarcoma and adjacent normal tissues. Adjacent normal tissues and different stages of osteosarcoma tissues (I–II and III stage) were stained with anti-Ki67 (red) and anti-KDM5A (green) antibodies. In the right peak diagram, fluorescent intensity of Ki67 and KDM5A signals across the regions marked with white arrows were shown in line plots, each peak represents the expression of Ki67-positive (Ki67+, red) and KDM5A (KDM5A+, green). Scale bar: 50 μm. **B**, **C** Relative Ki67 and KDM5A expression in normal tissues and osteosarcoma tissues of I–II sand III stage were measured by calculating the IntDen/area using ImageJ 1.52a. Expression levels were normalized to DAPI. **D** Histogram chart showing the percentage of KDM5A+/Ki67+ double-positive cells between adjacent normal and tumor tissues. The differences between groups were assessed using unpaired *t* test for continuous variables and analysis of variance (ANOVA), *p < 0.05, **p < 0.001.

KDM5A showed a similar higher level of mRNA expression in sarcomas (SARC) (Fig. 1A) compared to some tumors like breast-invasive carcinoma (BRCA), kidney renal clear-cell carcinoma (KIRC), glioblastoma multiforme (GBM), kidney renal papillary cell carcinoma (KIBP), brain lower-grade glioma (LGG), and ovarian serous cystadenocarcinoma (OV)^18^. Kaplan–Meier survival analysis^19^ with autoselect best cutoff was performed and showed that SARC patients with higher expression of KDM5A had a shorter relapse-free survival (RFS) time (Fig. 1B). Since it is obvious that KDM5A has a significant causal or strongly prognostic effect on SARC, so, the expression level of KDM5A in collected twenty-one paired osteosarcoma and adjacent normal tissues (Enneking staging, I–II stage: *n* = 10, III stage: *n* = 11) was detected and evaluated by using immunohistochemistry (IHC) staining (Fig. 1C). Accordingly, to evaluate the expression level, KDM5A was significantly upregulated in osteosarcoma tissues compared with the adjacent normal tissues (Fig. 1D). Subsequently, the expression of the cell division marker Ki67, indicative of tumor cell proliferation, was subjected to analysis whether the increase in KDM5A expression was related to tumor cell growth (Fig. 2A). We observed the percentage of KDM5A-positive (KDM5A^+^) (Fig. 2B), Ki67-positive (Ki67^+^) (Fig. 2C), and KDM5A^+^/Ki67^+^ double-positive (Fig. 2D) cells in the malignant osteosarcoma group was significantly higher than in the normal group, which was correlated with Enneking stages. These results suggest that upregulation of the expression of KDM5A contributes to cell growth of human osteosarcoma.

### Knockdown of KDM5A resulted in a decrease in osteosarcoma cell proliferation

**Fig. 3:**
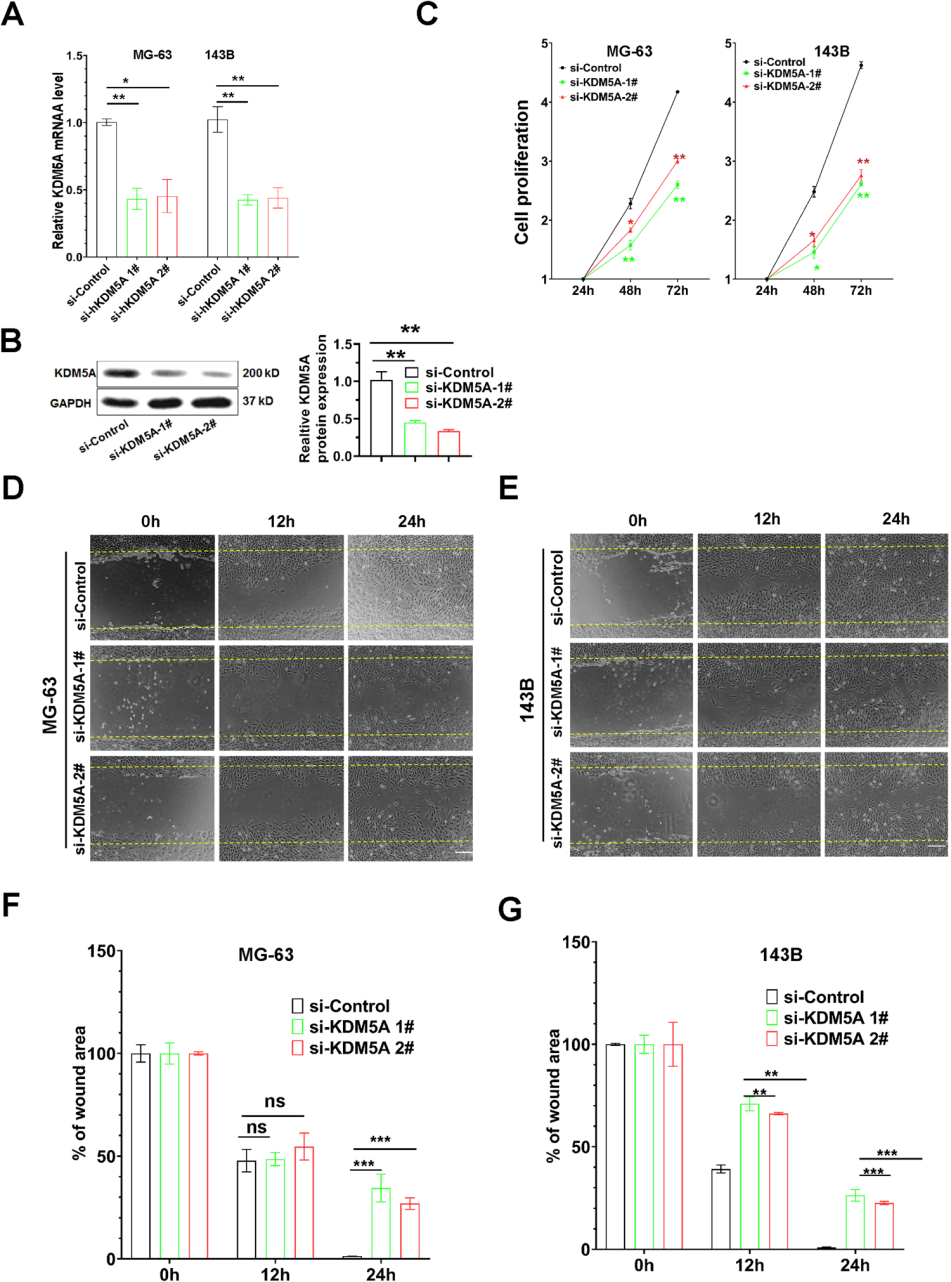
Knockdown of KDM5A reduced osteosarcoma cell proliferation. **A** RT-qPCR analysis showed the expression levels of KDM5A in MG-63 and 143B cells with KDM5A knockdown after 48 h (hour). Expression levels were normalized to GAPDH. si-Control, scramble small-interfering RNA; si-KDM5A, small-interfering KDM5A. **B** Knockdown efficiency of KDM5A in 143B cells was assessed by western blot. GAPDH served as the internal loading control. **C** The CCK-8 assay was performed to evaluate the proliferation of MG-63 and 143B cells with KDM5A knockdown after 48 h and 72 h. Proliferation rate was normalized to the value at 24 h. **D**, **E** The wound-healing assay showed the wound area of MG-63 and 143B cells with KDM5A knockdown after 12 and 24 h. **F**, **G** Analysis of the wound-healing area of MG-63 and 143B cells with KDM5A knockdown. The wound-healing area was normalized to the value at 0 h. All data were shown as the mean ± S.D (*n* = 3). *p < 0.05, **p < 0.01, and ***p < 0.001. ns, no significant.

To test our finding demonstrated above, we performed knockdown of the KDM5A gene in MG-63 and 143B cells by transfection with two siRNAs, which has higher knockdown efficiency^20^. The transfection efficiency of siRNA-KDM5A (si-KDM5A) in MG-63 and 143B cells was confirmed by using RT-qPCR assays (Fig. 3A) and western blot (Fig. 3B). The CCK-8 assay revealed that knockdown of KDM5A significantly suppressed the growth of MG-63 and 143B cells (Fig. 3C). Furthermore, the wound-healing assay showed that the areas of the scratch wound in the si-KDM5A groups were significantly larger than in the si-control group in MG-63 (Fig. 3D, E) and 143B cells (Fig. 3F, G). These results validated that KDM5A promotes the proliferation of osteosarcoma cells.

### Knockdown of KDM5A induced cell-cycle slowdown and promoted apoptosis

**Fig. 4:**
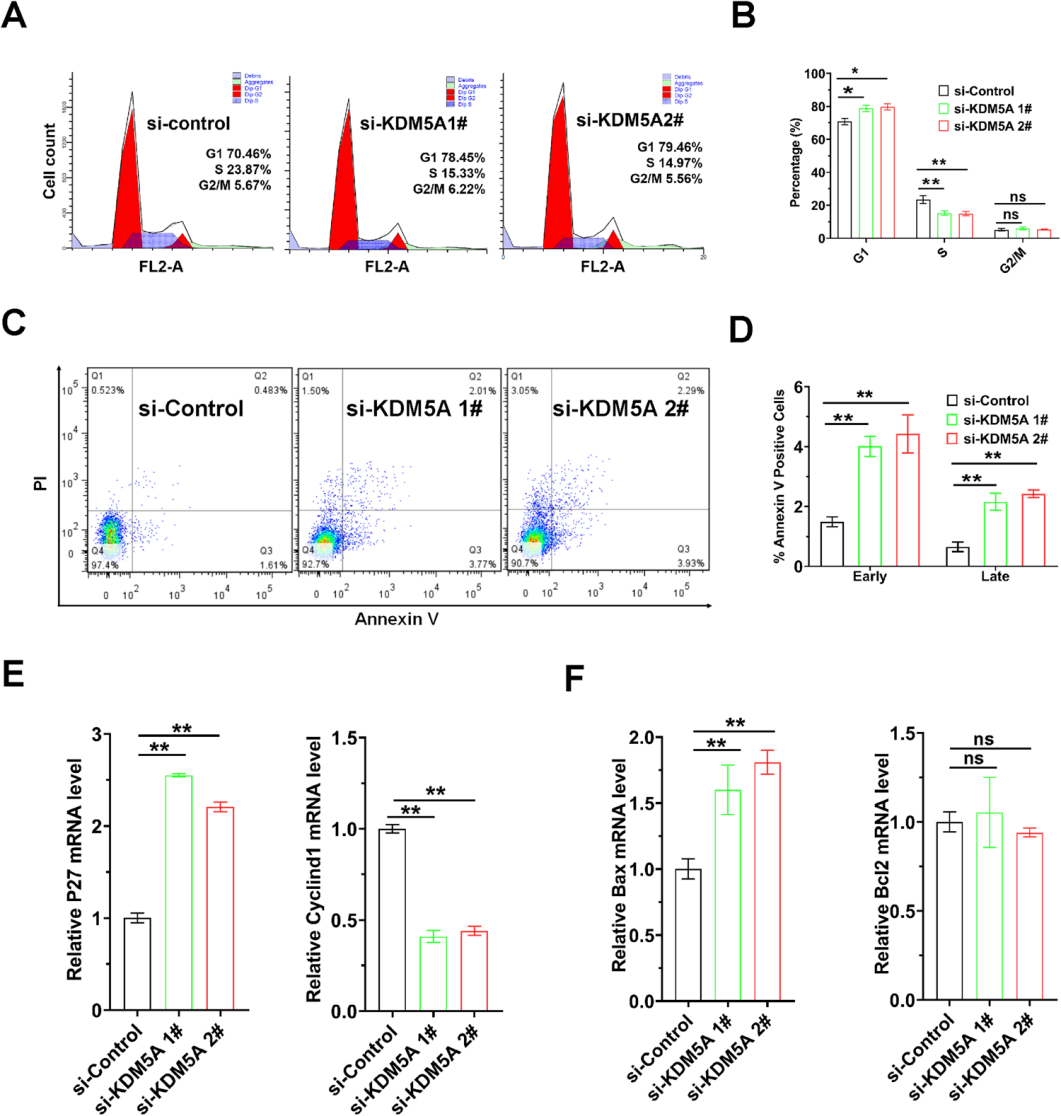
KDM5A knockdown induced cell-cycle arrest and promoted apoptosis. **A** Flow cytometry images of the cell cycle in 143B cells with KDM5A knockdown. The percentage of cells in G1, S, and G2/M phases was analyzed using ModFitLT software. **B** The quantified cell-cycle results were shown as a percentage of the total cells. **C** FACS analysis was used to detect apoptotic cells stained with Annexin V-APC/propidium iodide (PI). **D** The percentage of Annexin V-positive apoptotic cells was quantified. **E** qRT-PCR analysis of the expression of cell-cycle-related genes P27 and Cyclin D1 in 143B cells with KDM5A knockdown after 48 h. **F** qRT-PCR analysis of the expression of apoptosis-related genes Bax and Bcl2 in 143B cells with KDM5A knockdown after 48 h. All data were shown as the mean ± S.D (*n* = 3). *p < 0.05, **p < 0.01. ns, no significant.

Flow cytometry cell-cycle analysis showed decreased S-phase ratio after KDM5A knockdown and demonstrated that KDM5A affects cell-cycle progression (Fig. 4A, B). The expression of the apoptosis marker Annexin V was increased following knockdown of KDM5A (Fig. 4C, D). Furthermore, RT-qPCR revealed that KDM5A knockdown increased the expression of cell-cycle inhibitor cyclin-dependent kinase inhibitor 1B (CDKN1B, also named as P27) expression and decreased Cyclin D1 expression (Fig. 4E), which is consistent with a previous report^21^. RT-qPCR revealed that apoptotic factor Bax was upregulated after siRNA-mediated depletion of KDM5A (Fig. 4F). These results suggest that KDM5A enhances the proliferation through promoting cell-cycle progression and inhibiting apoptosis in osteosarcoma cells.

### Knockout of KDM5A suppressed the tumorigenesis of osteosarcoma cells in vivo

**Fig. 5:**
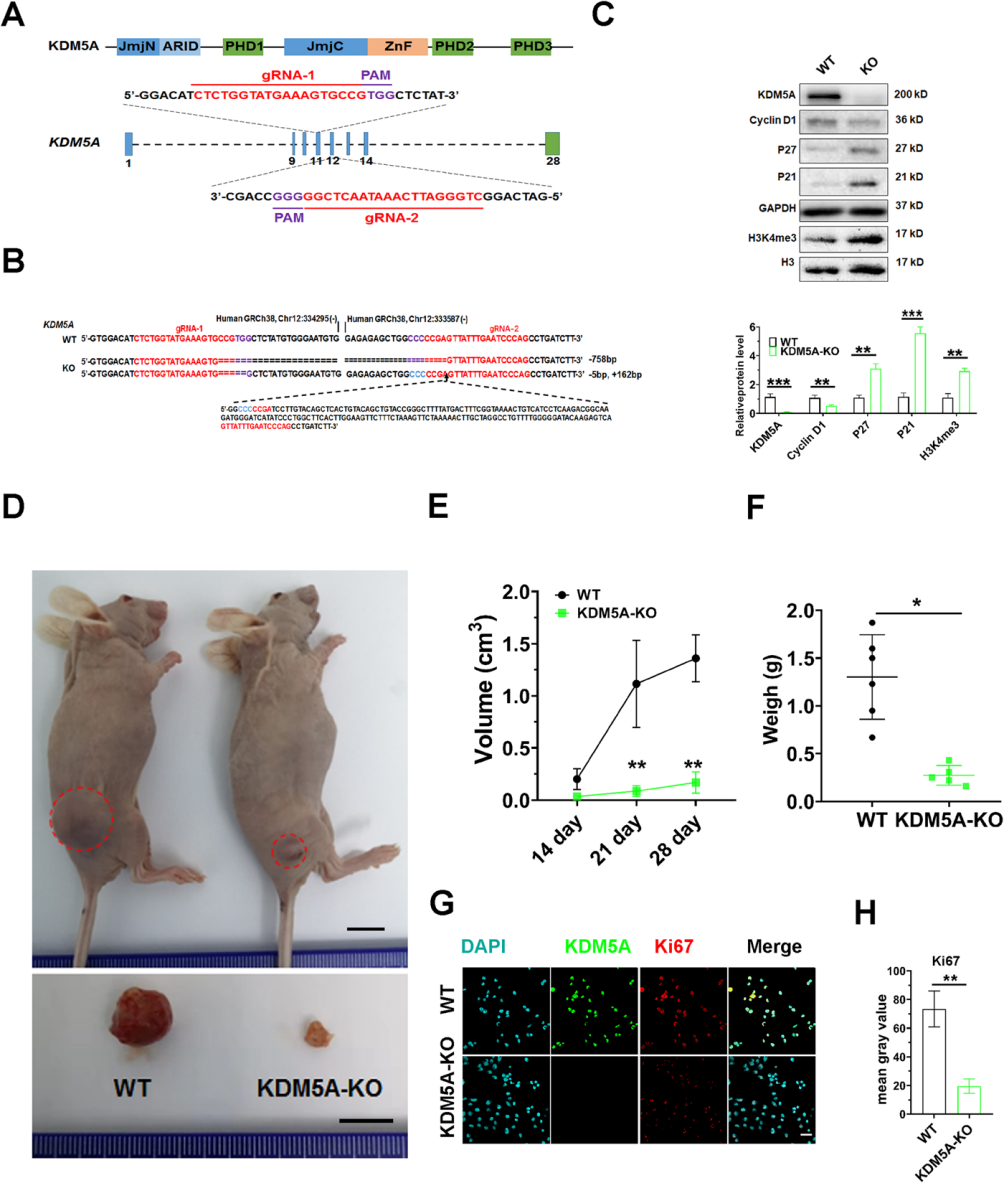
KDM5A knockout reduced tumor growth in vivo. **A** Strategy to create KDM5A-KO cell lines by CRISPR/Cas9 system in 143B cell line. The KDM5A protein domain architecture was shown on the upper panel. The exon information was shown in the lower panel. The sgRNA-targeting sequences of exons 11 and 12 encoding the catalytic domain JmjC motif of KDM5A were labeled in red, and the protospacer-adjacent motif (PAM) sequence is labeled in purple. **B** The DNA sequencing of both alleles of targeted sequences in one mutant clone. A 758-bp deletion was detected in one allele, and 5-bp deletion and 162-bp insertion in another allele. **C** The expression levels of KDM5A, Cyclin D1, P27, P21, and H3K4me3 in KDM5A-KO cell line were detected by western blot analysis. GAPDH as the internal loading control for Cyclin D1, P27, P21, and H3 for H3K4me3. **D** KDM5A-KO and 143B cells were respectively injected subcutaneously into the right side of the abdomen of the nude mice (1 × 10^6^ cells per mouse, *n* = 6). The tumor-bearing mice were collected at day 28. The representative photo of mice and gross tumors in the mice were shown. Scale bar: 1 cm. **E** The tumor volume in each group was measured once a week. **F** The weight of the tumors in each group. **G** Co-immunofluorescent staining of DAPI (blue), KDM5A (green), and Ki67 (gray) on 143B and KDM5A-KO tumor specimens. Scale bar: 50 µm. **H** Relative Ki67 expression in 143B and KDM5A-KO tumor tissues was measured by calculating the IntDen/area using ImageJ 1.52a. Statistical analysis was conducted using a *t* test, except in (**H**), where one-way ANOVA with Dunnett post test was used. *p < 0.05, **p < 0.01.

In order to evaluate the potential effect of KDM5A on osteosarcoma cell proliferation in vivo, the CRISPR/Cas9 system was used to knock out KDM5A in 143B cell lines (Fig. 5A). PCR sequencing showed that both alleles were mutated in one cell clone named KDM5A-KO (Fig. 5B). Western blot of KDM5A confirmed the knockout efficiency in the KDM5A-KO cell line (Fig. 5C). The suppressing growth (Fig. S1A), decreased S-phase ratio (Fig. S1B, S1C), and increased apoptosis (Fig. S1D and S1E) were also observed after KDM5A knockout. Furthermore, RT-qPCR and western blot revealed that KDM5A knockout increased P27 and P21 expression and decreased Cyclin D1 expression (Fig. 5C and Fig. S1F). The apoptotic factor Bax was also upregulated after KDM5A knockout (Fig. S1F). KDM5A was a histone demethylase specific for demethylating trimethylated lysine 4 of histone H3 (H3K4me3)^22,23^. Therefore, the upregulation of H3K4me3 was detected in KDM5A-KO cells (Fig. 5C). Wild-type (WT) 143B and KDM5A-KO cells were subcutaneously inoculated into nude mice, respectively (day 0). The sizes of the tumors were measured once a week and the tumors were separated from nude mice at day 28 (Fig. 5D). Weights and sizes of tumors in the KDM5A-KO group were lighter and smaller than those in the WT group; thus, KDM5A knockout could suppress tumor growth in vivo (Fig. 5E, F). Quantitative IHC analysis showed that the expression of Ki67 was decreased in the xenograft tumors of the KDM5A-KO group (Fig. 5G, H). These results suggested that KDM5A enhances the proliferation through promoting cell-cycle progression and inhibiting apoptosis in osteosarcoma cells. These experiments confirmed the above results in vitro that siRNA-mediated KDM5A knockdown exerted its antigrowth activity by inhibiting cell proliferation.

### Transcriptome signatures associated with KDM5A in osteosarcoma

**Fig. 6:**
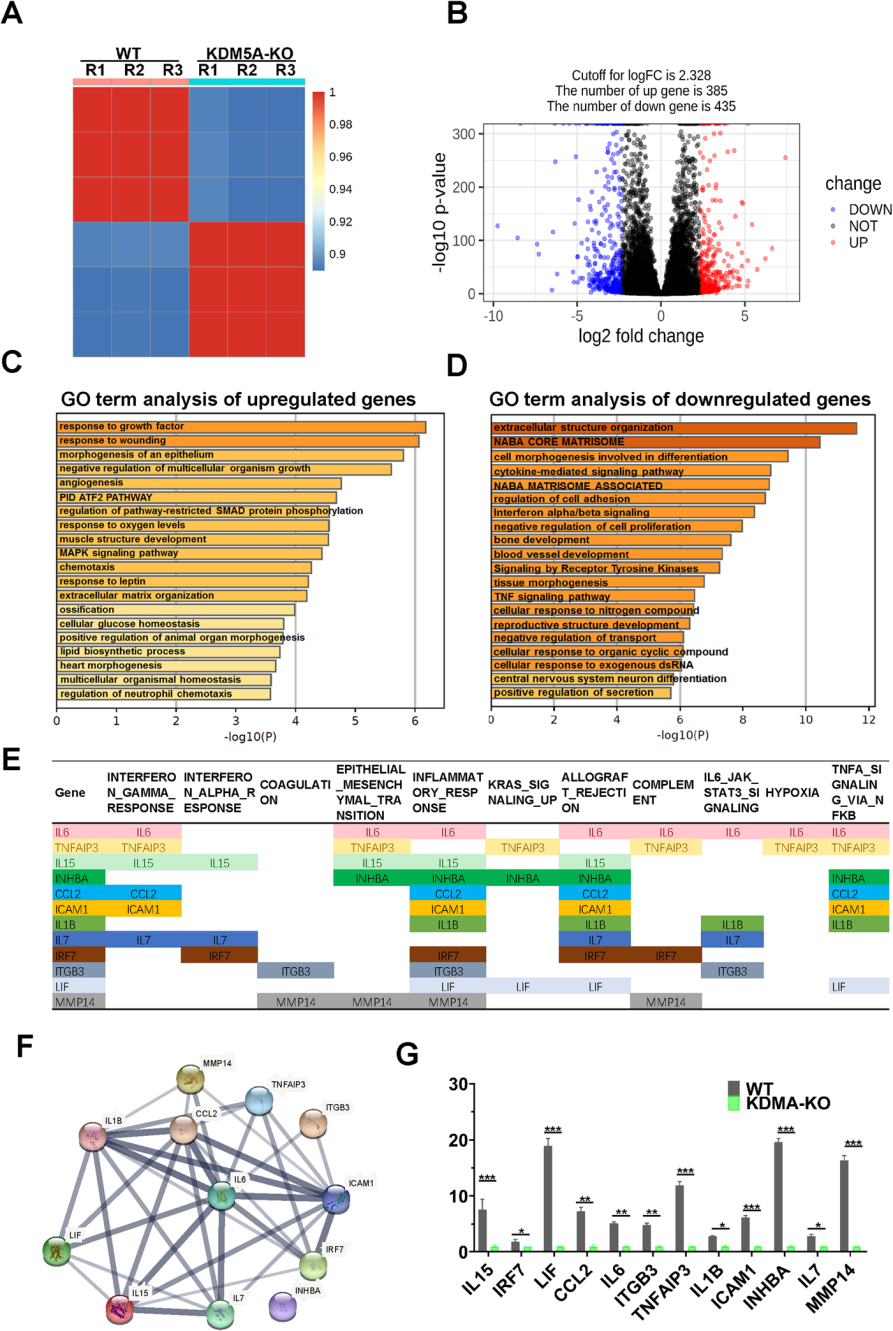
The transcriptome of KDM5A-KO cells. **A** Hierarchical clustering of gene expression profiles from 3 biologically independent samples based on Pearson correlation coefficient in WT and KDM5A-KO cells. Colors from green to red indicate weak-to- strong correlation. WT: wild type, 143B cell line. **B** Volcano plot of all genes analyzed in the present study. Blue, significantly differently downregulation genes. Red, significantly differently overregulation genes. Black, nonsignificantly differently expressed genes (DEG). **C**, **D** GO analysis of upregulated (**C**) and downregulated genes (**D**) for biological processes in KDM5A-KO cells. **E** The genes that were involved in >4 pathways of enrichment sets, including IL6 (shared by eight pathways), TNFAIP3 (shared by six pathways), IL15 and INHBA (shared by five pathways), CCL2, ICMA1, IL1B, IL7, IRF7, ITGB3, LIF, and MMP14 (shared by four pathways). All core enrichment genes in the 13 enriched sets were shown in Supplementary Table S1. **F** A protein–protein interaction (PPI) network of the 12 enrichment genes was constructed by using the STRING online database (https://string-db.org/). **G** The expression of the enrichment genes in KDM5A-KO cells was detected using RT-qPCR analyses. Data were shown as the mean ± S.D (*n* = 3). *p < 0.05, **p < 0.01, ***p < 0.01.

In order to elucidate the mechanism underlying the antiproliferative effect of KDM5A in osteosarcoma, we carried out the transcriptome analysis on KDM5A-KO cells. KDM5A-KO cells exhibited a slightly different expression pattern compared with 143B cells (Fig. 6A and Fig S2). Additionally, a volcano plot was constructed to examine differentially expressed genes (DEG) expression distributions, including 385 upregulated and 435 downregulated genes (Fig. 6B). Gene ontology (GO) analysis indicated that genes related to negative regulation of multicellular organism growth were upregulated (Fig. 6C), and “extracellular structure organization”, “NABA CORE MATRISOME”, and “NABA MATRISOME ASSOCIATED” were downregulated (Fig. 6D) in KDM5A-KO cells. To identify biological pathways associated with KDM5A expression in osteosarcoma, we performed gene set enrichment analysis (GSEA) on the transcriptome dataset and observed that 50 gene sets were different between 143B and KDM5A-KO cells, and 13 sets were enriched in 143B cells (FDR < 0.25, *p* *<* 0.05) (Fig. S3). Among the enrichment sets, some networks were commonly related to proliferation of osteosarcoma, such as epithelial–mesenchymal transition (EMT)^24^, IL6/JAK/STAT3^25^, KARS^26^, and TNF-α/NF-κB pathway^27^. Among these sets (Table S1), there were 12 genes that are involved in >4 pathways, including IL6 (shared by eight pathways), TNFAIP3 (shared by six pathways), IL15 and INHBA (shared by five pathways), and CCL2, ICMA1, IL1B, IL7, IRF7, ITGB3, LIF, and MMP14 (shared by four pathways) (Fig. 6E). STRING *PPI* analysis showed remarkably significant interactions among those genes (Fig. 6F). RT-qPCR analyses validated significant downregulation of expression of each of these genes in KDM5A-KO cells (Fig. 6G). These findings suggest that KDM5A participates in osteosarcoma proliferation through the activation of various signaling pathways involved in cellular growth.

## Discussion

The present work illustrates that KDM5A is a potential oncogene in osteosarcoma via analysis of publicly available datasets, clinical subjects, and functional experiments. First, we found that KDM5A was upregulated in osteosarcoma tissues than the adjacent normal tissues. Furthermore, the expression level of KDM5A was associated with osteosarcoma cell proliferation and tumor tumorigenesis. We also found that decreased osteosarcoma cell proliferation induced by KDM5A deficiency was associated with downregulation of cyclin D1 and upregulation of p27, P21, and apoptosis regulator Bax. KDM5A was well known in many types of human cancer pathogenesis via promotion of cell growth, inhibition of tumor suppressor gene expression, and development of drug tolerance^28^. Thus, overexpression of KDM5A in osteosarcoma promotes cell proliferation by inducing cell-cycle-related genes and inhibiting apoptosis.

Next, GO analysis and GSEA of KDM5A-KO-related transcriptome indicated that KDM5A activates multiple signaling pathways, including EMT, IL6/JAK/STAT3, and TNF-α/NF-κB to promote proliferation. These pathways have been reported to play an important role in the osteosarcoma progression^24–27^. As the gene involved in the largest number signaling, interleukin-6 (IL6) as a multifunctional cytokine, promoted glycolytic metabolism and significantly increased proliferation, colony formation of, and promoting an EMT-like phenotype of osteosarcoma cells^25^. Cinobufagin inhibited osteosarcoma cell proliferation and tumorigenesis capability via blocking IL6–OPN–STAT3 signaling pathway^29^. Tumor necrosis factor alpha-induced protein 3 (TNFAIP3), also known as A20, was a ubiquitin-editing enzyme that promotes melanoma cell proliferation in vitro and melanoma growth in vivo through the overexpression of cyclin D and phosphor Rb^30^. IL6 also increased the expression of intercellular adhesion molecule-1 (ICAM-1) and promoted migration in human osteosarcoma cells^31^. Previous studies revealed that TNF-α axis pathway was activated in human osteosarcoma cells^27^. The inhibition of the TNF-α/NF-κB pathway led to the accumulation of p21^Cip1^, thereby inducing cell-cycle arrest at the S phase^32^. The decreased S-phase ratio and Cyclin D1 expression and increased P27 and P21 expression after KDM5A deficiency was also detected in our research. Therefore, KDM5A promotes cell proliferation through these multiple pathways. The exact roles and mechanism need to be investigated in future research.

KDM5A, a tri- and dimethylated lysine 4 of histone H3 (H3K4me3/2)-specific histone demethylase^15,16^, belonged to JARID1 family^33^. This family is well known to regulate normal cell fates during development and contributes to the epigenetic abnormalities that underlie malignant transformation^22,34–36^. We also detected the increased expression of H3K4me3 in KDM5A-KO osteosarcoma cells (Fig. 5C). In estrogen receptor (ER)-positive breast cancer, KDM5A is key regulator of phenotypic heterogeneity and inhibition of the enzyme activity increases sensitivity to endocrine therapy^34^. Cellular phenotypic heterogeneity was a key mechanism underlying tumor progression and therapeutic resistance^37^. Therefore, KDM5A as an oncogene in osteosarcoma through the function of histone demethylase needs further study.

More interestingly, some downregulated clusters were mainly enriched in ‘extracellular structure organization’, ‘NABA CORE MATRISOME’, and ‘NABA MATRISOME ASSOCIATED’ in GO analysis (Fig. 6D). Core matrisome (CM), the extracellular matrix (ECM) protein inventory, serves functions ranging from cellular adhesion and motility to cell signaling^38^. ECM played an important role in tumor migration and invasion through the cytoskeleton changing and undergoing EMT^39^. TGF-β/BMP2 signaling pathways also promoted osteosarcoma cell migration and invasion^40^. Moreover, TNFAIP3 could potentiate the invasive and migratory capacities of melanoma cells in vitro and melanoma metastasis in vivo by promoting epithelial–mesenchymal transition (EMT)^30^. IL6 enhanced lung colonization of OS cells by overexpression of ICAM-1 and promoted tumor metastasis^41^. TGF-α/EGFR interacted PI3K/Akt activation, then activated NF-κB, increased the expression of ICAM-1, and contributed to the migration of human osteosarcoma cells^42^. Therefore, KDM5A may also promote the metastasis of osteosarcoma.

In conclusion, we have demonstrated that the expression of KDM5A was increased in osteosarcoma tissues, and reduced KDM5A level could decrease osteosarcoma cell growth in vitro and prevent osteosarcoma tumorigenesis in vivo. These results suggest that KDM5A may be a potential molecular biomarker and novel target for osteosarcoma therapy.

## Materials and methods

### Patients and tissue samples

Twenty-one osteosarcoma specimens and matched adjacent normal tissues used in this study were collected from the Department of Spinal Surgery, Hospital (T.C.M) Affiliated to Southwest Medical University. This study was approved by the Ethics Committee of Southwest Medical University and written informed consent was obtained from all the patients. All procedures performed in this work were conducted in ethical standards and principles of the 1964 Helsinki Declaration and its later amendments^43^.

### Cell culture and reagents

The osteosarcoma cell lines (143B and MG-63) were purchased from the *National Infrastructure of Cell Line Resource* (China) and cultured in Dulbecco’s modified Eagle’s medium (Gibco, 11965-092) supplemented with 10% fetal bovine serum (Hyclone, SH 30396.03). All cell lines used in this work were maintained at 37 °C with 5% CO_2_ and humidified environment. Cells were dissociated with 0.05% Trypsin-EDTA (Gibco, 25300-062) at 37 °C for 3 min. The cell numbers were counted using the Scepter^TM^ cytometer (Millipore). Antibodies were purchased from Abcam (Cambridge, UK) and Cell Signaling Technology (Danvers, USA).

### Oligonucleotides and transfection

For KDM5A RNA interference (RNAi), the single silencing RNA (siRNA) of KDM5A (si-KDM5A) and its control siRNA (si-Control) were purchased from GenePharma and used to transfect the MG-63 and 143B cells for knockdown KDM5A expression. For siRNA transfection, 1 × 10^3^ MG-63 or 143B cells were seeded into 96-well plates at day 0. At day 1, these cells were transfected with si-KDM5A or si-Control at a 50 nM concentration with RNAiMAX Transfection Reagent (Invitrogen). The transfection efficiency of knockdown was assessed using quantitative RT-PCR (RT-qPCR) and western blot (WB). The sequences of the siRNAs used in this study were listed in Supplementary Table S2.

### Quantitative reverse transcription PCR

RNA was extracted using TRIzol reagent according to the manufacturer’s recommended protocol (Invitrogen). About 1 μg of total RNA was reverse transcribed into first-strand cDNA using the PrimeScript 1st Strand cDNA Synthesis Kit (TaKaRa, Japan). RT-qPCR was performed in triplicate using Fast SYBR^®^ Green Master Mix (TaKaRa, Japan). As a loading control, GAPDH was used for each gene. The primers used in this study were listed in Supplementary Table S2.

### Cell counting kit-8 (CKK-8) assay

Cell proliferation was assessed using CCK-8 (CK04-01, DOJINDO, Japan) assay. The cells were seeded into 96-well plate, and 10 μL of the CCK-8 solution was then added to each well at the indicated times. The absorbance at 450 nm and 600 nm was measured using the iMark^TM^ microplate reader (Bio-Rad, Hercules, USA).

### Immunohistochemistry and immunofluorescence

Tumor tissues were dissected, collected, and fixed in 10% phosphate-buffered formalin (1:10 tissue–formalin ratio) overnight at 4 °C. Then, Paraffin sections were performed according to standard protocols. Then, representative tumor areas of the paraffin-embedded specimens were incubated with the primary antibodies and the appropriate Alexa Fluor-coupled secondary antibodies.

Cells were fixed with 4% paraformaldehyde and permeablized with 0.5% Triton X-100 for 10 min, then blocked with 3% BSA for 1 h at room temperature (RT). Afterward, the cells were treated with their primary antibody overnight at 4 °C, rinsed three times with PBS, followed by incubating with secondary antibody for 1 h at RT. Subsequently, DAPI was used to label the cell nuclei. The following antibodies were used for immunohistochemistry (IHC) and immunofluorescence (IF): anti-KDM5A (3876, Cell Signaling Technology), anti-H3K4me3 (abcam, ab185637), and anti-Ki67 (abcam, ab15580). The results of IF were quantified using ImageJ software. The images of stained slides were photographed with a confocal fluorescence microscope (Olympus, FV3000, Japan).

### Quantitative immunofluorescence

Quantitative immunofluorescence (QIF) was performed following standard protocols designed in the previous studies^44^. Briefly, the QIF of each channel was quantified as the mean fluorescence intensity on the desired region from the slides containing the tumor specimens or cells. All images were captured with the same exposure time, allowing subsequent QIF analysis in the respective channel to be comparable. For all imaging analyses in this study, two to four independent experiments were performed and >10 cells were selected for QIF analysis in each experiment. Plots shown in figures were from one representative experiment.

### Western blot

Whole-cell lysates were separated by sodium dodecyl sulfate (SDS)-polyacrylamide gel electrophoresis (SDS-PAGE), and transferred to polyvinylidene difluoride (PVDF) membranes (Millipore, USA). The membranes were blocked in Tris-buffered saline containing 5% BSA and 0.1% Tween-20 for 1 h at RT, and then incubated with the primary antibody overnight at 4 °C. The member was treated with a horseradish peroxidase-conjugated secondary antibody for 1 h at RT. Immunoreactive bands were visualized using enhanced chemoluminescence and the ChemiDoc XRS molecular imager (Bio-Rad, USA). The antibodies used in this study were listed in Supplementary Table S1.

### CRISPR/Cas9-mediated KDM5A knockout in 143B cells

sgRNA-specifying oligo sequences spanning exons 11 and 12 of KDM5A were chosen to minimize the potential off-target mutagenesis based on publicly online tools (http://www.e-crisp.org/E-CRISP/). These annealed oligo sequences were cloned into the vector pX330 (Addgene, #42230) to construct two plasmids, pX330–KDM5A–gRNA1 and pX330–KDM5A–gRNA2. These two plasmids and a puromycin-resistant plasmid were co-electroporated into 143B cells using Neon Transfection System (Invitrogen). After 48 h of puromycin selection, electroporated cells were trypsinized and seeded at very low density. On day 14, about one hundred cell colonies were picked up manually and expanded. Genomic DNA was extracted from these cells, and the mutant colonies were assayed by PCR amplification and Sanger sequencing using specific primers spanning the deletion region. The oligoes and primers used in CRISPR/Cas9 were listed in Supplementary Table S2.

### In vivo tumor xenotransplantation

For subcutaneous models, wild-type (WT) 143B cells and KDM5A-KO cells were suspended in PBS, and 1 × 10^6^ cells (100 μL) were injected subcutaneously into nude mice. Tumor size was measured every week, and tumor volumes were calculated with the following formula: volume = *(a* × *b*^*2*^*)/2*, in which *a* is the longest diameter and *b* is the shortest diameter. Mice were euthanized to collect tumors when tumor diameter reached 1.5 cm. All animal care and mice experimental procedures were approved by the Institutional Animal Care and Use Committee at Hospital (T.C.M) Affiliated to Southwest Medical University. All the mice used in this work were bred and maintained in a specific pathogen-free (SPF) environment.

### RNA-sequencing analysis

All RNA-sequencing (RNA-Seq) reads were mapped to UCSC mouse genome (mm10) using Hisat2 (version 2.1.0) with default parameters. Read counts per gene were determined using feature Counts (Version 1.6.4) and differential gene expression analysis was performed using DESeq2. Genes with FDR < 0.05 and fold change (FC) > 1.5 were considered to be differentially expressed genes (DEG). To measure gene expression level, FPKM (fragments per kilobase of exon per million mapped reads) of genes was normalized against total uniquely mapped reads and exon length. Pearson correlation coefficients between each of the two samples were calculated using function cor() in R, and the matrix of Pearson correlation coefficients was used to generate the heatmap using R package pheatmap. Z score-normalized FPKM values were used for gene clustering by R package pheatmap^45^. Gene ontology (GO) analysis was conducted by R package cluster Profiler. Gene set enrichment analysis (GSEA) was used by comparing the knockout group to the WT control by java GSEA Desktop Application (Version 4.0.3), which was downloaded from the GSEA website (http://www.broadinstitute.org/gsea/). The parameters used were as follows: Gene set database, h.all.v7.2.symbols.gmt [Hallmarks]; Number of permutations, 1000; Phenotype labels, KO vs WT; Gene symbols, No_Collapse; Permutation type, phenotype.

### Statistical analysis

The data were shown as the means ± S.D unless stated otherwise. A two-tailed Student’s *t* test was used to analyze significant differences. QIF signal differences between groups were assessed using unpaired *t* test for continuous variables and analysis of variance (ANOVA). *p* < 0.05 was considered as significant (*p < 0.05, **p < 0.01, ***p < 0.001). For GSEA, *p* values were corrected using the Benjamini–Hochberg procedure, with the resulting *q* value considered a measure of false discovery rate (FDR). GraphPad Prism v8.0.1 was used to analyze and arrange the data.

## Supplementary information

Supplementary Figure S1

Supplementary Figure S2

Supplementary Figure S3

Supplementary Table S1

Supplementary Table S2
