# Supplementary figures and images for: Histone demethylase KDM5A promotes tumorigenesis of osteosarcoma tumor
Source: Cell Death Discov. 2021 Jan 12;7:9. doi: 10.1038/s41420-020-00396-7 (PMC7803953; doi:10.1038/s41420-020-00396-7)

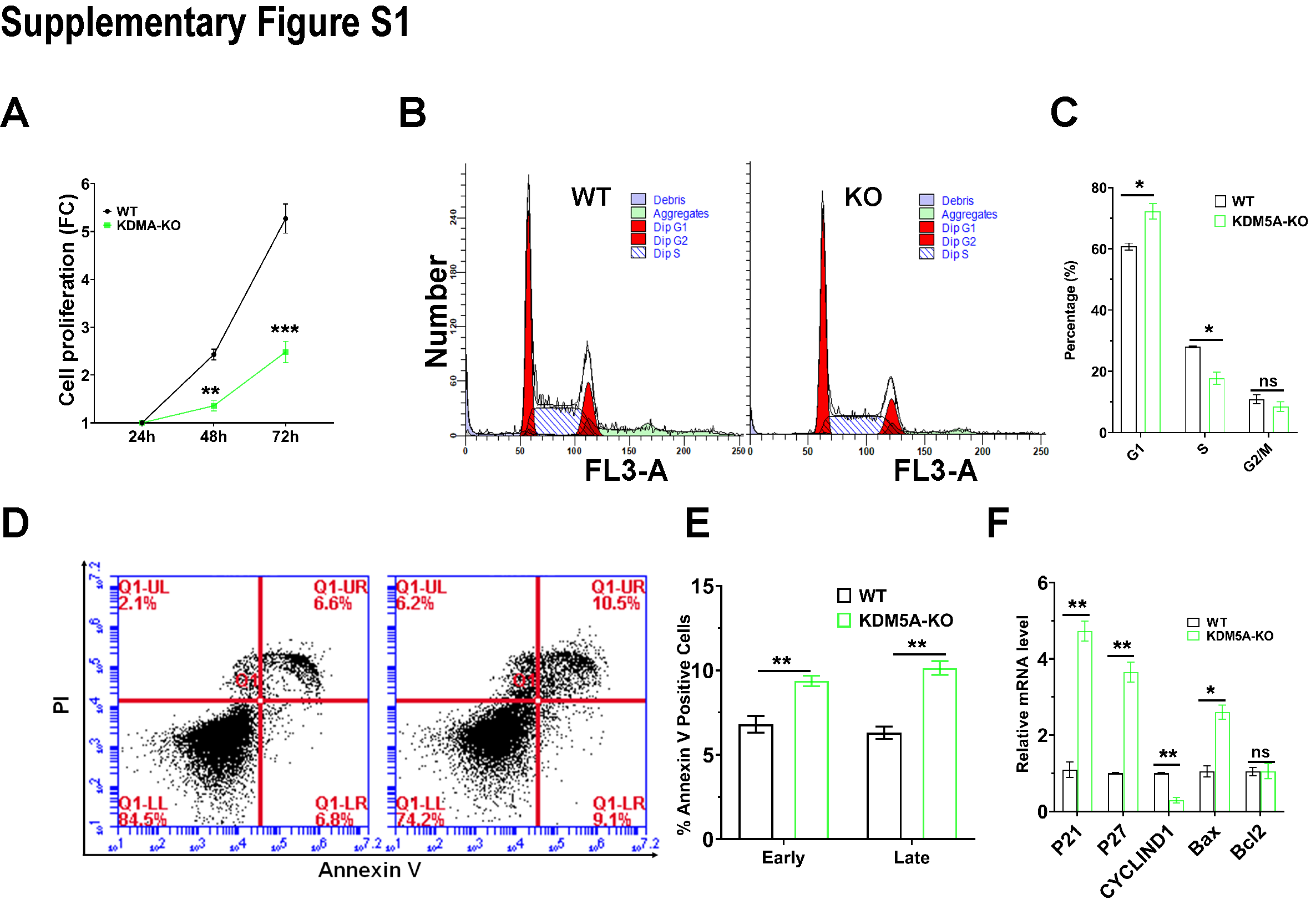

Supplement: Supplementary file 1 — Supplementary Figure S1 [file 41420_2020_396_MOESM1_ESM.tif]

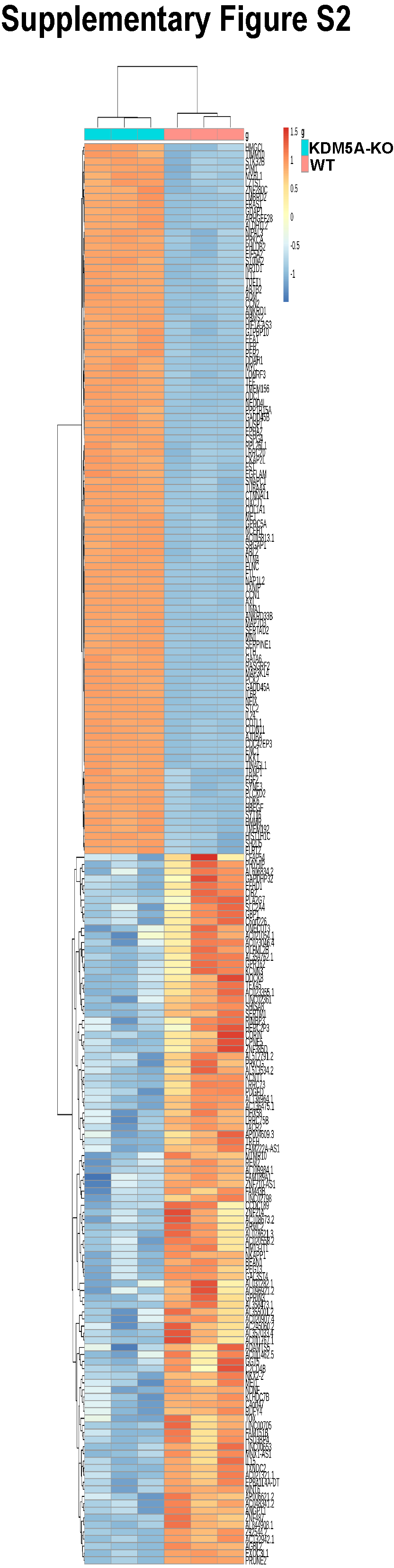

Supplement: Supplementary file 2 — Supplementary Figure S2 [file 41420_2020_396_MOESM2_ESM.tif]

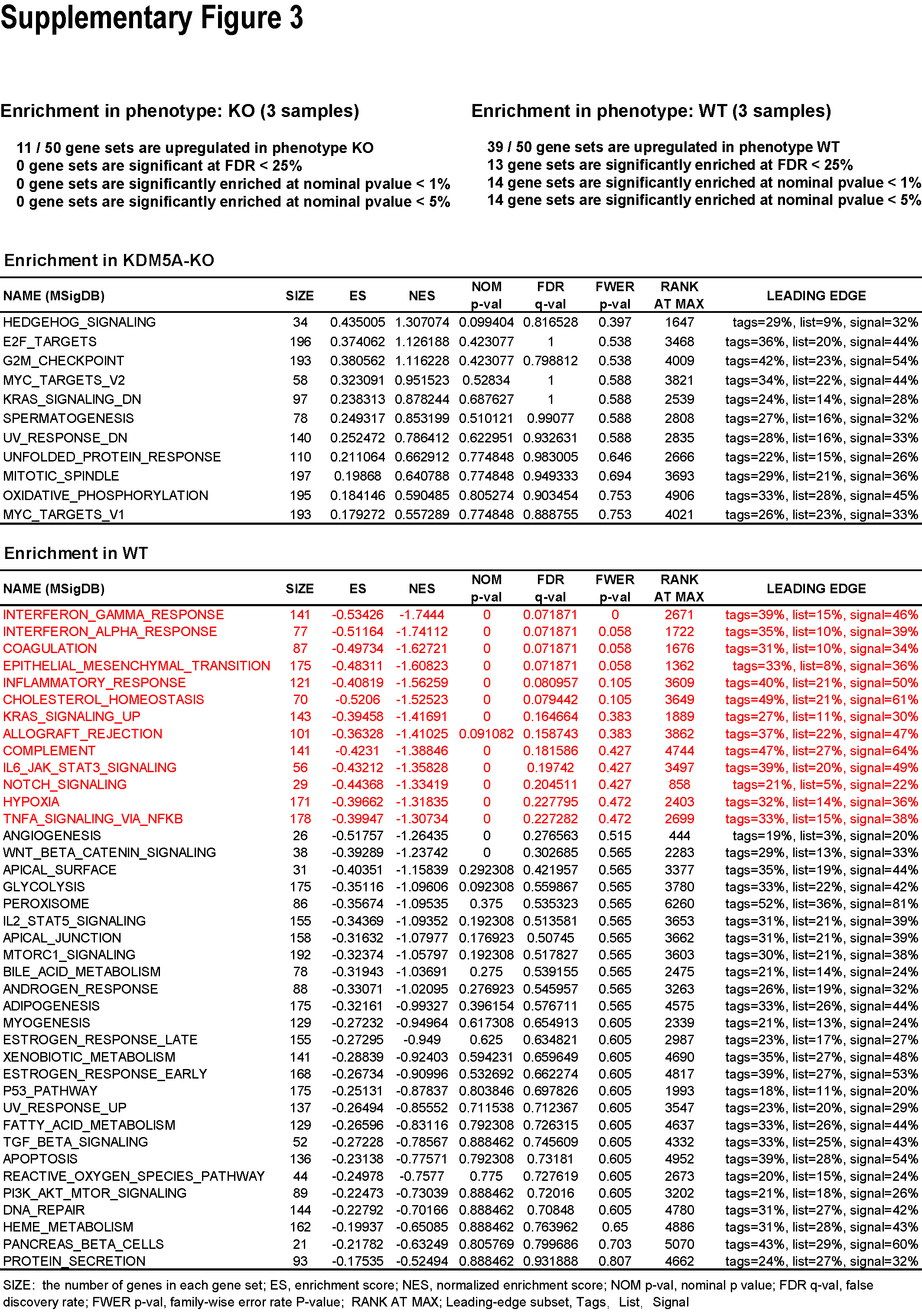

Supplement: Supplementary file 3 — Supplementary Figure S3 [file 41420_2020_396_MOESM3_ESM.tif]
